# RNA-Seq Transcriptome Analysis of Direction-Selective T4/T5 Neurons in *Drosophila*

**DOI:** 10.1371/journal.pone.0163986

**Published:** 2016-09-29

**Authors:** Katarina Pankova, Alexander Borst

**Affiliations:** 1 Max Planck Institute of Neurobiology, Martinsried, Germany; 2 Graduate School of Systemic Neurosciences, LMU Munich, Munich, Germany; EPFL, SWITZERLAND

## Abstract

Neuronal computation underlying detection of visual motion has been studied for more than a half-century. In *Drosophila*, direction-selective T4/T5 neurons show supralinear signal amplification in response to stimuli moving in their preferred direction, in agreement with the prediction made by the Hassenstein-Reichardt detector. Nevertheless, the molecular mechanism explaining how the Hassenstein-Reichardt model is implemented in T4/T5 cells has not been identified yet. In the present study, we utilized cell type-specific transcriptome profiling with RNA-seq to obtain a complete gene expression profile of T4/T5 neurons. We analyzed the expression of genes that affect neuronal computational properties and can underlie the molecular implementation of the core features of the Hassenstein-Reichardt model to the dendrites of T4/T5 neurons. Furthermore, we used the acquired RNA-seq data to examine the neurotransmitter system used by T4/T5 neurons. Surprisingly, we observed co-expression of the cholinergic markers and the vesicular GABA transporter in T4/T5 neurons. We verified the previously undetected expression of vesicular GABA transporter in T4/T5 cells using VGAT-LexA knock-in line. The provided gene expression dataset can serve as a useful source for studying the properties of direction-selective T4/T5 neurons on the molecular level.

## Introduction

Processing of visual cues and detecting the direction of motion in particular is critical for the survival of many organisms. In *Drosophila*, visual motion processing begins at the level of photoreceptors in retina that use histamine as their neurotransmitter [1]. Photoreceptor signals are segregated into parallel ON- and OFF-channels represented at the cellular level by glutamatergic L1 neurons (ON-channel) and cholinergic L2 neurons (OFF-channel) [2,3,4,5]. The identified downstream components of the ON motion vision pathway are Mi1 and Tm3 neurons which synapse on the T4 neurons [6,7,8,9]. In the OFF motion vision pathway the L2 neurons provide input to Tm1, Tm2, Tm4 and Tm9 cells that in turn connect with T5 neurons [4,7,8,10,11,12]. The neurotransmitter used by Tm1, Tm2, and Tm9 cells is acetylcholine [4,11]. In other cell types presynaptic to T4 and T5 neurons, the neurotransmitter systems have not been characterized yet.

Within each column of the fly visual system, four subtypes of the T4 and T5 neurons exist which signal the local direction of motion along one of the four orthogonal directions and project accordingly into one of the four layers of the lobula plate [13,14]. T4/T5 cells are the elementary motion detectors and blocking of their synaptic transmission results in a complete loss of the fly optomotor response [15,16]. Since none of the cells presynaptic to T4/T5 cells are directionally selective [7,8,10,12], the dendrites of the T4/T5 neurons represent the first processing stage in the fly visual system where the direction of image motion is explicitly represented [13,14].

A theoretical model for direction selectivity proposed by Hassenstein and Reichardt [17] has been dominating the field of insect motion vision. The Hassenstein-Reichardt (HR) model consists of a coincidence detector that receives input from two adjacent points in space. Signal propagation in one of these input channels is temporally delayed resulting in simultaneous arrival of the signals to the coincidence detector only when the luminance moves across space in a particular direction and velocity. Subsequently, the synchronously arriving signals become amplified in the coincidence detector in a multiplicative way. HR model explains an enhancement of signals moving along the preferred direction of T4/T5 neurons [14] whereas an additional inhibitory mechanism causes suppression of signals moving in the opposite, so-called ‘null-direction’ of the cells [18,19].

**Fig 1:**
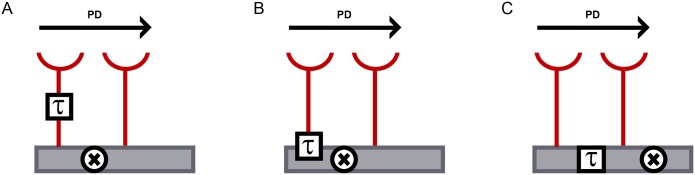
Implementation of the time delay in HR model. Schematics of a direction-selective dendrite (grey rectangle) receiving input from two channels (depicted in red). Visual stimulus moving in a preferred direction (PD) causes sequential activation of the two input channels. Due to temporal delay (τ) in signal propagation in one of the input channels, the two signals triggered by a PD stimulus reach the dendritic mechanism for supralinear amplification (shown as ‘X’) simultaneously and are as result summed in a non-linear fashion. Visual stimuli moving in other directions do not lead to signal amplification in the dendrite.

While current work in the field refines the algorithmic structure of the mechanism leading to direction selectivity in T4/T5 neurons and maps different presynaptic cell types onto the emerging circuit, the biophysical implementation of the two key processes, i.e. temporal delay and nonlinear signal-interaction, remains largely unknown. Regarding the origin of the temporal delay between two input signals to the HR detector, three mutually not exclusive scenarios have been suggested [20]: 1.) The temporal delay between two input arms is generated presynaptically to T4/T5 neurons (Fig 1A). 2.) The temporal delay arises at the level of neurotransmitter receptors on the dendrites of T4/T5 neurons (Fig 1B). 3.) Dendritic filtering creates a temporal offset between the input signals within the dendrites of T4/T5 cells (Fig 1C). The biophysical implementation of the other critical feature of the HR model, the supralinear summation of coinciding signals, represents an open question as well. The following mechanisms have already been demonstrated to underlie supralinear summation in different neurons of other species: activation of voltage-gated sodium and/or calcium channels [21,22], activation of NMDA receptors [23], opening of rectifying electrical synapses [24] and deactivation of inhibitory currents [25]. As the present knowledge about expression of genes that could determine the computational properties of T4/T5 neurons is limited [11], the implementation of the HR model of direction-selectivity at the molecular level on the dendrites of T4/T5 neurons is still an unresolved issue.

Here, we provide a complete transcriptional profile of all protein-encoding genes in T4/T5 neurons. We examine expression levels of the identified neurotransmitter receptors and gap junction proteins in order to characterize the input T4/T5 neurons receive from their presynaptic partners. Furthermore, we analyze the expression of voltage-gated and non-gated ion channels that may underlie the mechanism for coincidence detection in the T4/T5 neurons. Unexpectedly, our RNA-seq data reveals that T4/T5 neurons co-express vesicular transporters for acetylcholine and GABA. In addition to cholinergic markers, T4/T5 neurons also produce GABA-degrading enzyme GABA transaminase. We confirm the co-expression of the vesicular GABA transporter (VGAT) and acetylcholine-synthesizing enzyme ChAT in all T4/T5 neurons using the transgenic VGAT-LexA knock-in line (Simpson 2016; J Neurogenetics, in revision) and immunostaining with antibody against ChAT.

## Materials and Methods

### Fly stocks

Flies were raised on standard cornmeal-agar food at 25°C. The following stocks were used: 10xUAS-unc84::2xGFP (provided by F. Schnorrer) [26], GMRSS00324 (R59E08-AD; R42F06-DBD) (provided by A. Nern) [27], R42F06-Gal4 (provided by G. Rubin) [15], UAS-mCD8::GFP (BDSC #5130) [28], VGAT-LexA (provided by J. Simpson) (Simpson 2016; J Neurogenetics, in revision) and LexAop-myr::mCherry (provided by B. Dickson) [29].

### Immunoprecipitation of nuclei

The following protocol was modified from the previously described procedure [26]. 300 μl of Dynabeads Protein G magnetic beads (Thermo Fisher Scientific) were incubated with 10 μl of the monoclonal anti-GFP antibody (Sigma-Aldrich, G6539) for 30 minutes at 4°C. Afterwards, beads were washed three times with 0.1% PBT. Approximately 50–60 ml of flies with the genotype w^-^; R59E08-AD/+; R42F06-DBD/10xUAS-UNC-84::2xGFP were anesthetized by CO_2_ and frozen in liquid nitrogen. Flies were decapitated by vigorous vortexing. Heads were smashed using Dounce grinder (Sigma-Aldrich) with loose pestle in ice-cold buffer (10 mM β-glycerophosphate, 2 mM MgCl2, 0.5% Igepal). The homogenate was passed through a 180 μm nylon net filter (Millipore) and filtrate was smashed again in Dounce grinder with tight pestle. After additional filtering through a 20 μm nylon net filter (Millipore), the homogenate was brought to 50 ml with sucrose buffer (10 mM β-glycerophosphate, 2 mM MgCl2, 25 mM KCl, 250 mM sucrose) and the antibody-preincubated magnetic beads were added. The binding reaction was carried out at 4°C for 30 min and was followed by five washes of the bead-bound nuclei with sucrose buffer. The bead-bound nuclei were imaged on a Leica TCS SP8 laser-scanning confocal microscope with DAPI (1 μg/ml) as a nuclear marker. In total, 636 DAPI-positive nuclei were manually counted, out of which 597 (94%) were GFP-positive.

### RNA isolation and RNA-seq

RNA from bead-bound nuclei was extracted with Trizol reagent (Thermo Fisher Scientific). Total RNA from two biological replicates (0.7 μg and 0.8 μg) was submitted to EMBL Genomics Core Facility, Heidelberg, Germany. A cDNA library was generated using TruSeq Stranded mRNA LT Sample Prep Kit (Illumina) and single-end sequenced on Illumina HiSeq 2000 to 51 bp read length. TopHat (v2.1.0) [30] was used to align untrimmed reads to the annotated genome of *D*. *melanogaster* (FlyBase r6.04). Alignment was carried out with default settings, except for excluding reads that mapped to more than one genomic position. In two biological replicates, 90% and 87% of total reads were uniquely mapped, resulting in a total of 154 and 119 million aligned reads. Reads mapping to gene exons were counted with the ‘featureCounts’ software (Rsubread package v1.12.6) [31] in R (v3.0.2). Read counts per gene were normalized by total exon length of a gene and the sum of reads assigned to all exons to generate the reads per kilobase per million reads mapped (RPKM) values (S1 Table).

### Immunohistochemistry

Fly brains were dissected in PBS and fixed in 4% PFA with 0.1% TritonX for 25 minutes. Brains were washed in 0.3% PBT and incubated first with primary (24-72h) and then secondary (24-48h) antibodies in 0.3% PBT supplemented with 5% NGS at 4°C. Brains were mounted in Vectashield mounting medium (Vector Laboratories) and imaged on Leica TCS SP5 laser-scanning confocal microscope. We used the following antibodies: rabbit anti-GFP (Torrey Pines, 1:400), mouse anti-nc82 (DSHB, deposited by E. Buchner, 1:200), rat anti-RFP, (Chromotek 5F8, 1:50), mouse anti-ChAT (DSHB, deposited by P. Salvaterra, 1:50), goat anti-rabbit Alexa 488 (Thermo Fisher Scientific, 1:200), goat anti-rat Alexa 568 (Thermo Fisher Scientific, 1:200) and goat anti-mouse Alexa 647 (Thermo Fisher Scientific, 1:200).

## Results

### RNA-seq data

**Fig 2:**
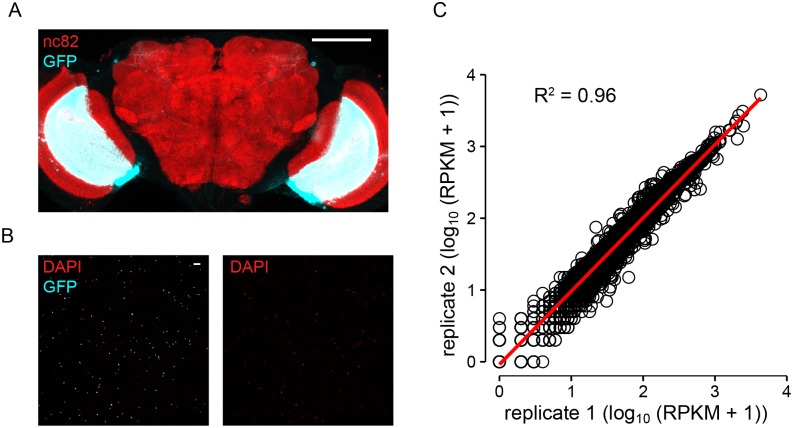
Specificity and reproducibility of RNA-Seq. (A) Expression pattern of the split Gal4 line labelling specifically T4/T5 neurons. In the central brain, the expression is—with an exception of a single pair of neurons—absent. The fly genotype was w^-^; R59E08-AD/+; R42F06-DBD/UAS-mCD8::GFP. The anti-GFP staining is shown in cyan and anti-nc82 staining is in red. Scale bar: 100 μm. (B) The immunoprecipitated GFP-tagged nuclei (cyan) were labelled with DAPI (red) to quantify the number of nuclei without GFP expression and as a result, the proportion of nuclei belonging to cells other than T4/T5. Scale bar: 30 μm. (C) Correlation of RPKM values of the two biological replicates plotted on a logarithmic scale. Linear regression (red line) accounts for 96% of the variation among the two replicates.

We performed RNA-seq of the mRNA extracted from immunoprecipitated GFP-tagged nuclei of T4/T5 neurons [26]. Cell type-specificity of our approach was achieved by using a split Gal4 line with expression restricted to T4/T5 neurons [27] (Fig 2A) and the high purity (94%) of the isolated GFP-labelled nuclei (Fig 2B). Two independent biological replicates showed high correlation of their expression values (Fig 2C), confirming the reproducibility of the obtained RNA-seq results. The genome-wide expression levels in T4/T5 neurons were within the range 0–4770 RPKM (S1 Table). We plotted all analyzed expression levels on an arbitrary color scale with the minimum at 0 and maximum at 100 RPKM, arguing that 95% of the genes in T4/T5 neurons have an expression value within these limits.

### Neurotransmitter receptors and gap junction proteins in T4/T5 neurons

**Fig 3:**
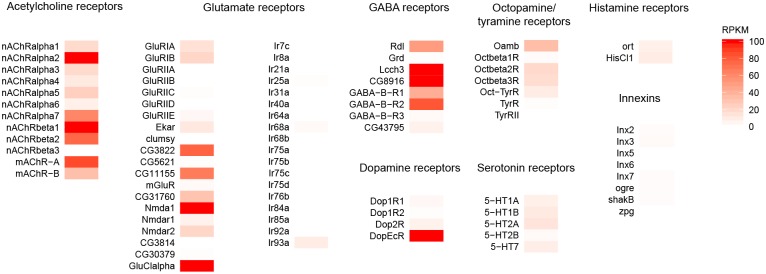
Expression of neurotransmitter receptors and gap junction proteins in T4/T5 neurons. Gene expression levels of the identified receptors for acetylcholine, glutamate, GABA, dopamine, octopamine, tyramine, serotonin and histamine as well as the innexin proteins are plotted as mean RPKM values using a color scale with the minimum at 0 and maximum at 100 RPKM.

We analyzed expression of the identified membrane receptors for all known neurotransmitters in *Drosophila* (Fig 3). In addition, we considered the possibility that T4/T5 neurons may receive input via electrical synapses and, therefore, examined expression of the known gap junction proteins as well (Fig 3). RNA-seq results showed that four subunits of nicotinic acetylcholine receptor (Dα2, Dα7, Dβ1 and Dβ2) had high expression levels. In addition, the muscarinic acetylcholine receptor mAChR-A was strongly expressed, while the other muscarinic receptor, mAChR-B, showed lower expression levels. Among the identified glutamate receptors, we observed strong expression of the glutamate-gated chloride channel GluClα which has been shown to mediate the hyperpolarizing action of glutamate in fly neurons [32,33,34]. We detected high expression levels of the ionotropic glutamate receptor subunits CG3822 and CG11155. Despite the very weak expression of the functional subunits of the NMDA receptor Nmdar1 and Nmdar2, we found that a related gene, Nmda1 that encodes a protein associated with the NMDA receptor is strongly expressed. Nmda1 gene has not been fully described yet and it is not clear whether it has functional role in neurotransmission.

Our RNA-Seq results showed presence of transcripts also for ionotropic GABA receptor subunits Rdl, Lcch3 and CG8916 as well as metabotropic GABA receptors GABA-B-R1 and GABA-B-R2. None of the known receptors for serotonin, histamine or tyramine were expressed in T4/T5 neurons. Amongst the octopamine receptors, we detected low expression of Oamb, suggesting weak octopaminergic input to T4/T5 neurons. Dopamine-ecdysone receptor DopEcR was expressed at high levels in T4/T5 neurons. Nevertheless, presence of DopEcR does not necessarily imply that T4/T5 neurons receive dopaminergic input as DopEcR also serves as a detector of steroid hormones [35]. We did not detect expression of any gap junction proteins of the innexin family in the T4/T5 neurons.

### Ion channels in T4/T5 neurons

**Fig 4:**
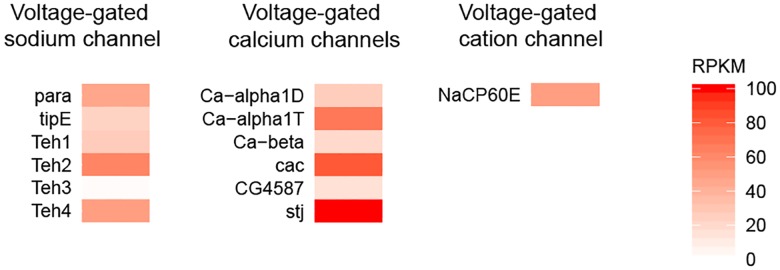
Expression of voltage-gated sodium and calcium channels in T4/T5 neurons. RPKM values of the identified structural and modulatory subunits of voltage-gated sodium and calcium channels are plotted on a color scale ranging from 0 to 100 RPKM.

The whole *Drosophila* genome contains only one gene that encodes a voltage-gated sodium channel—named para—and five genes encoding auxiliary subunits that are known to modulate the gating of para. We found the voltage-gated sodium channel para to be expressed in T4/T5 neurons together with four out of the five identified modulatory subunits (Fig 4). All of the identified subunits of voltage-gated calcium channels were expressed in T4/T5 neurons as well (Fig 4). In addition, we also detected expression of the voltage-gated cation channel NaCP60E that is permeable for both, calcium and sodium (Fig 4).

**Fig 5:**
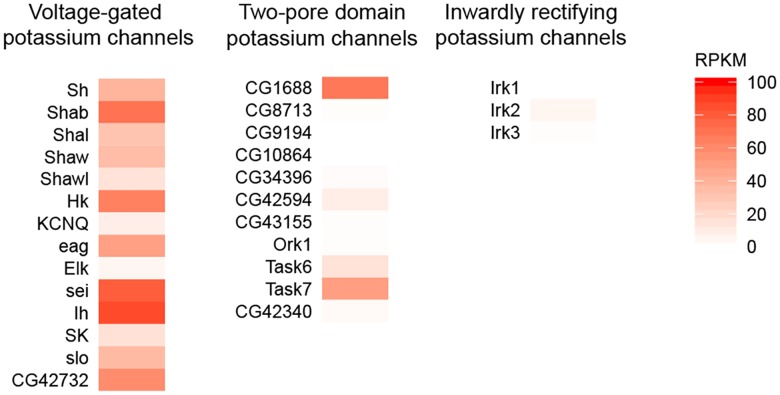
Expression of potassium channels in T4/T5 neurons. Expression levels of the identified members of the voltage-gated potassium channels, two-pore domain potassium channels and inwardly rectifying potassium channels are plotted as RPKM values on a color scale ranging from 0 to 100 RPKM.

Potassium channels can be divided based on their structure and function into three main groups: voltage-gated potassium channels, two-pore domain potassium channels and inwardly rectifying potassium channels. With the exception of Elk and KCNQ, all of the identified members of voltage-gated potassium channels were expressed in T4/T5 neurons (Fig 5). Expression of Shawl and SK was rather weak (Fig 5). Two-pore domain potassium channels have been shown to mediate leak potassium current as well as chemo- and mechano-sensation [36]. In T4/T5 neurons, two members of this family, CG1688 and Task7 were strongly expressed (Fig 5). None of the inwardly rectifying potassium channels was present in T4/T5 neurons (Fig 5).

### Neurotransmitter phenotype of T4/T5 neurons

**Fig 6:**
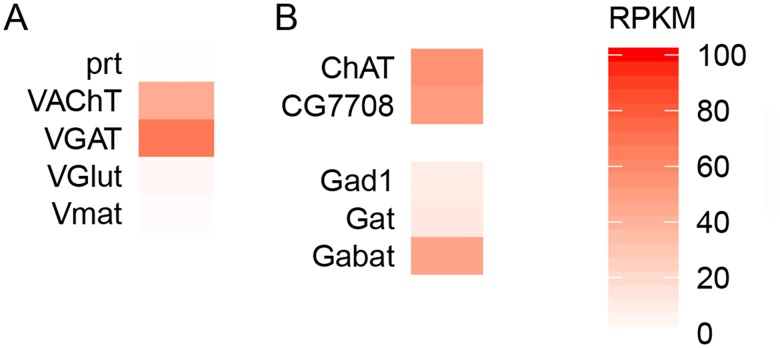
Neurotransmitter phenotype of T4/T5 neurons. Expression values of the identified vesicular neurotransmitter transporters (A) and known markers of the cholinergic and GABAergic neurons (B) are plotted as RPKM levels on a color scale capturing the range 0–100 RPKM.

T4/T5 neurons have previously been shown to use acetylcholine as their neurotransmitter [11,33]. Hence, our expectation was to confirm the cholinergic phenotype of these neurons. Indeed, we found the genes for the acetylcholine-synthesizing enzyme ChAT and the vesicular acetylcholine transporter (VAChT) to be expressed at high levels (Fig 6). Surprisingly, however, we also found the gene for the vesicular GABA transporter (VGAT) to be expressed in T4/T5 neurons (Fig 6A).

**Fig 7:**
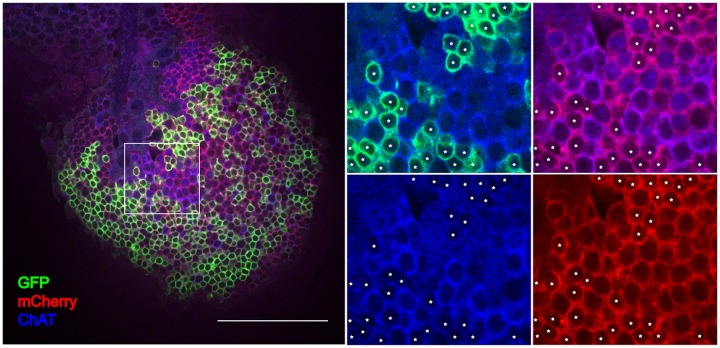
Co-expression of ChAT and VGAT in T4/T5 neurons. Optic lobe of a fly with genotype w^-^; VGAT-LexA/LexAop-myr::mCherry; R42F06-Gal4/UAS-mCD8::GFP was immunostained with antibodies against GFP (green), mCherry (red) and ChAT (blue). Boxed area in the left picture is enlarged in four panels in the right. White asterisks mark the position of the GFP-labelled cell bodies of the T4/T5 neurons. Somatic membrane of the T4/T5 neurons co-localizes with anti-mCherry as well as with anti-ChAT immunostaining. Scale bar: 50 μm.

To prove the expression of VGAT in T4/T5 neurons by another line of evidence, we used a transgenic fly line VGAT-LexA that had the sequence for bacterial transcription factor LexA inserted into the first exon of the VGAT gene (Simpson 2016; J Neurogenetics, in revision). Utilizing two binary expression systems, Gal4/UAS [37] and LexA/lexAop [38], we labelled T4/T5 neurons with GFP and cells with an active VGAT locus with mCherry. The immunostaining revealed that T4/T5 neurons were indeed part of the VGAT-LexA expression pattern (Fig 7). The apparent co-expression of VAChT and VGAT in T4/T5 neurons raises the possibility that T4/T5 neurons compose two different populations; one cholinergic and one expressing VGAT. To test this, we performed further staining against acetylcholine-synthesizing enzyme ChAT that showed that all of the VGAT-expressing T4 and T5 neurons were in addition cholinergic as well (Fig 7).

Besides the vesicular transporter VAChT and acetylcholine-synthesizing enzyme ChAT, cholinergic neurons are characterized by the presence of the membrane choline transporter that is in *Drosophila* encoded by the gene CG7708. Our RNA-seq results confirmed the expression of all three cholinergic markers in the T4/T5 neurons (Fig 6). On the other hand, GABAergic neurons are characterized by the presence of GABA-synthesizing enzyme glutamate decarboxylase (Gad1), membrane GABA transporter (Gat) and GABA-degrading enzyme GABA transaminase (Gabat). We found that T4/T5 neurons did not express Gad1 and Gat but their expression of Gabat was comparable to that of cholinergic markers (Fig 6), suggesting that degradation of GABA is taking place in T4/T5 neurons.

## Discussion

### Comparison of expression data obtained with RNA-seq and RT-PCR

A low-throughput transcript profiling of selected 22 genes in T4/T5 neurons was already performed previously using RT-PCR amplification of mRNA from the isolated somata of either T4 or T5 neurons [11]. Shinomiya et al. [11] detected expression of ChAT and potassium channel slo in both, T4 and T5 neurons, muscarinic acetylcholine receptor mAChR-A in T5 cells and mAChR-B in T4 cells, in agreement with our results (Figs 3, 5 and 6). However, transcripts for nicotinic subunit Dβ3 identified by RT-PCR in T4/T5 neurons [11] were not detected in our RNA-seq dataset (Fig 3). On the other hand, we observed strong expression of nicotinic receptor subunits Dα2, Dβ1, Dβ2 (Fig 3) and vesicular transporter VGAT (Fig 6) while none of these genes was identified as expressed in the RT-PCR assay [11]. These discrepancies are likely due to lower detection threshold caused by smaller amounts of input mRNA used in reported RT-PCR experiments and higher risk of contamination of the analyzed neurons with other cell types during the manual sampling of somata.

Our transcriptome analysis with RNA-seq pools T4 and T5 cells together as there is no available Gal4 line with the expression pattern restricted exclusively to either T4 or T5 neurons. The Gal4 lines used previously to target either T4 or T5 neurons [11,13,33] show expression also in other neuronal types in the central brain, therefore they are not suitable for the approach used in this study that involves immunoprecipitation of neuronal nuclei from the whole fly heads. As a consequence of the pooling of T4 and T5 cells together in our analysis, genes that are differentially expressed in T4 and T5 neurons might show weaker expression in our dataset although their expression is strong but restricted to either T4 or T5 cells. In addition, T4/T5 neurons comprise a total of eight subtypes that differ in their preferred direction, orientation of their dendrites and projection pattern of their axons. Although all these cell types likely perform the same dendritic computations, this diversity might still affect our transcriptome data.

### Molecular implementation of HR model

A major contribution of our transcriptome analysis of T4/T5 neurons is narrowing down the biological mechanisms that can apply the HR model of motion detection to the dendrites of the T4/T5 neurons. Depending on where the time delay in the HR model arises (Fig 1), different molecular processes underlying coincidence detection come into question. Inputs that are temporally offset already presynaptically to the dendrites of the T4/T5 neurons (Fig 1A) can be summed in a supralinear fashion by rectifying electrical synapses [24], deactivation of Kir conductance [25] or voltage-gated sodium or calcium channels [21,22]. As neither the electrical synapses nor the inwardly rectifying potassium channels are present in the T4/T5 neurons (Figs 3 and 5), we suggest that voltage-gated sodium and calcium channels (Fig 4) are the main candidates for the supralinear summation in this scenario.

Our results show that T4/T5 neurons are likely to receive cholinergic, glutamatergic and GABAergic input (Fig 3). For each of these neurotransmitters, both ionotropic as well as G-protein coupled receptors are expressed in the T4/T5 neurons. As the ionotropic and metabotropic neurotransmitter receptors have different activation dynamics, the temporal delay can indeed be generated by the two inputs synapsing on different types of receptors on the dendrites of T4/T5 neurons (Fig 1B). Supralinear dendritic summation by NMDA receptors in the T4/T5 neurons is not likely as the expression levels of the functional NMDA receptor subunits are rather low (Fig 3). A coincidence detection mechanism involving deactivation of KCNQ-mediated hyperpolarizing potassium current via muscarinic acetylcholine receptor signaling cascade has already been proposed to take place in T4/T5 neurons [11,39]. However, the weak expression of KCNQ in T4/T5 neurons makes the contribution of KCNQ currents to a supralinear signal amplification in T4/T5 neurons unlikely (Fig 5). As a potential substrate for the recently discovered null-direction suppression [18,19], T4/T5 neurons express GABA receptors as well as glutamate-binding hyperpolarizing GluClα channel (Fig 3). Such inhibitory inputs could also shape the receptive field properties of T4/T5 neurons making them more sensitive to gratings orthogonal to their preferred orientation [14].

Dendritic signal propagation depends on the morphology of the dendrites as well as on their active and passive membrane properties. Possible differential temporal filtering in the dendritic compartments of T4/T5 cells (Fig 1C) can be caused by inhomogeneous distribution of ion channels mediating leak currents, low threshold potassium currents or hyperpolarization-activated potassium currents. We identify expression of candidate channels in the T4/T5 neurons that might mediate these currents such as leak potassium channels CG1688 and Task7, hyperpolarization-activated potassium channel Ih and low threshold-activated potassium channels Shab, Shaw [40], eag [41] and sei [42] (Fig 5).

### Co-existence of acetylcholine and GABA in T4 and T5 neurons?

GABAergic neurons in flies are traditionally characterized by the presence of glutamic acid decarboxylase Gad1, an enzyme responsible for GABA synthesis [43]. Mammalian neurons, however, can release GABA in absence of glutamate decarboxylase. This is achieved either by re-uptaking GABA from the extracellular space via GABA transporters [44] or by its synthesis with alternative pathways [45,46]. GABA transaminase (Gabat) can convert succinic semialdehyde into GABA and vice versa [47], and is thought to participate in the degradation of GABA in most cells, not in its synthesis [48]. In T4/T5 neurons, the Gad1 and Gat are expressed very weakly (Fig 6B) and therefore their contribution to potential GABAergic transmission of T4/T5 neurons is questionable. On the other hand, Gabat is expressed at the levels comparable to cholinergic markers (Fig 6B) suggesting that degradation of GABA is taking place in T4/T5 neurons. Alternatively, Gabat might be the GABA synthesizing enzyme in the T4/T5 neurons.

T4/T5 neurons synthesize and release acetylcholine from their axons onto their postsynaptic partners, the lobula plate tangential cells [11,33]. In addition to axon terminals, T4 neurons also possess presynaptic neurotransmitter release sites in their dendrites [6]. The identified recipients of the dendritic synaptic input from T4 neurons are Mi9, C3 and other T4 neurons [6]. The functional role of dendritic synapses of T4 neurons has not been investigated yet, and neither has it been shown that these synapses are cholinergic. For comparison, in mouse retina, direction-selective starburst amacrine cells (SACs) co-release acetylcholine and GABA [49] and GABAergic SAC-SAC connections shape the velocity tuning and contrast range of SACs [50]. Whether this could be the role of GABAergic transmission in T4/T5 neurons remains to be investigated. In flies, there has been no evidence provided so far for the release of more than one neurotransmitter from a single neuron.

## Supporting Information

S1 Table RPKM values in T4/T5 neurons. (XLSX)

## References

[1] Hardie RC. A histamine-activated chloride channel involved in neurotransmission at a photoreceptor synapse. Nature. 1989 6 29;339(6227):704–6. 10.1038/339704a0 2472552

[2] Joesch M, Schnell B, Raghu SV, Reiff DF, Borst A. ON and OFF pathways in Drosophila motion vision. Nature. 2010 11 11;468(7321):300–4. 10.1038/nature09545 21068841

[3] Eichner H, Joesch M, Schnell B, Reiff DF, Borst A. Internal structure of the fly elementary motion detector. Neuron. 2011 6 23;70(6):1155–64. 10.1016/j.neuron.2011.03.028 21689601

[4] Takemura SY, Karuppudurai T, Ting CY, Lu Z, Lee CH, Meinertzhagen IA. Cholinergic circuits integrate neighboring visual signals in a Drosophila motion detection pathway. Curr Biol. 2011 12 20;21(24):2077–84. 10.1016/j.cub.2011.10.053 22137471 PMC3265035

[5] Joesch M, Weber F, Eichner H, Borst A. Functional specialization of parallel motion detection circuits in the fly. J Neurosci. 2013 1 16;33(3):902–5. 10.1523/JNEUROSCI.3374-12.2013 23325229 PMC6704863

[6] Takemura SY, Bharioke A, Lu Z, Nern A, Vitaladevuni S, Rivlin PK, et al A visual motion detection circuit suggested by Drosophila connectomics. Nature. 2013 8 8;500(7461):175–81. 10.1038/nature12450 23925240 PMC3799980

[7] Behnia R, Clark DA, Carter AG, Clandinin TR, Desplan C. Processing properties of ON and OFF pathways for Drosophila motion detection. Nature. 2014 8 28;512(7515):427–30. 10.1038/nature13427 25043016 PMC4243710

[8] Strother JA, Nern A, Reiser MB. Direct observation of ON and OFF pathways in the Drosophila visual system. Curr Biol. 2014 5 5;24(9):976–83. 10.1016/j.cub.2014.03.017 24704075

[9] Ammer G, Leonhardt A, Bahl A, Dickson BJ, Borst A. Functional specialization of neural input elements to the Drosophila ON motion detector. Curr Biol. 2015 8 31;25(17):2247–53. 10.1016/j.cub.2015.07.014 26234212

[10] Meier M, Serbe E, Maisak MS, Haag J, Dickson BJ, Borst A. Neural circuit components of the Drosophila OFF motion vision pathway. Curr Biol. 2014 2 17;24(4):385–92. 10.1016/j.cub.2014.01.006 24508173

[11] Shinomiya K, Karuppudurai T, Lin TY, Lu Z, Lee CH, Meinertzhagen IA. Candidate neural substrates for off-edge motion detection in Drosophila. Curr Biol. 2014 5 19;24(10):1062–70. 10.1016/j.cub.2014.03.051 24768048 PMC4031294

[12] Serbe E, Meier M, Leonhardt A, Borst A. Comprehensive characterization of the major presynaptic elements to the Drosophila OFF motion detector. Neuron. 2016 2 17;89(4):829–41. 10.1016/j.neuron.2016.01.006 26853306

[13] Maisak MS, Haag J, Ammer G, Serbe E, Meier M, Leonhardt A, et al A directional tuning map of Drosophila elementary motion detectors. Nature. 2013 8 8;500(7461):212–6. 10.1038/nature12320 23925246

[14] Fisher YE, Silies M, Clandinin TR. Orientation selectivity sharpens motion detection in Drosophila. Neuron. 2015 10 21;88(2):390–402. 10.1016/j.neuron.2015.09.033 26456048 PMC4664581

[15] Schnell B, Raghu SV, Nern A, Borst A. Columnar cells necessary for motion responses of wide-field visual interneurons in Drosophila. J Comp Physiol A. 2012 5;198(5):389–95. 10.1007/s00359-012-0716-3 22411431 PMC3332379

[16] Bahl A, Ammer G, Schilling T, Borst A. Object tracking in motion-blind flies. Nat Neurosci. 2013 6;16(6):730–8. 10.1038/nn.3386 23624513

[17] Hassenstein V, Reichardt W. [System theoretical analysis of time, sequence and sign analysis of the motion perception of the snout-beetle Chlorophanus]. Z Naturforsch B. 1956; 11b:513–524. German.

[18] Haag J, Arenz A, Serbe E, Gabbiani F, Borst A. Complementary mechanisms create direction selectivity in the fly. Elife. 2016 8 9;5:e17421 10.7554/eLife.17421 27502554 PMC4978522

[19] Leong JC, Esch JJ, Poole B, Ganguli S, Clandinin TR. Direction selectivity in Drosophila emerges from preferred-direction enhancement and null-direction suppression. J Neurosci. 2016 8 3;36(31):8078–92. 10.1523/JNEUROSCI.1272-16.2016 27488629 PMC4971360

[20] Borst A, Helmstaedter M. Common circuit design in fly and mammalian motion vision. Nat Neurosci. 2015 8;18(8):1067–76. 10.1038/nn.4050 26120965

[21] Larkum ME, Zhu JJ, Sakmann B. A new cellular mechanism for coupling inputs arriving at different cortical layers. Nature. 1999 3 25;398(6725):338–41. 10.1038/18686 10192334

[22] Gabbiani F, Krapp HG, Koch C, Laurent G. Multiplicative computation in a visual neuron sensitive to looming. Nature. 2002 11 21;420(6913):320–4. 10.1038/nature01190 12447440

[23] Schiller J, Schiller Y, Clapham DE. NMDA receptors amplify calcium influx into dendritic spines during associative pre- and postsynaptic activation. Nat Neurosci. 1998 6;1(2):114–8. 10.1038/363 10195125

[24] Edwards DH, Yeh SR, Krasne FB. Neuronal coincidence detection by voltage-sensitive electrical synapses. Proc Natl Acad Sci U S A. 1998 6 9;95(12):7145–50. 10.1073/pnas.95.12.7145 9618553 PMC22768

[25] Wessel R, Kristan WB Jr, Kleinfeld D. Supralinear summation of synaptic inputs by an invertebrate neuron: dendritic gain is mediated by an "inward rectifier" K(+) current. J Neurosci. 1999 7 15;19(14):5875–88. 10407027 10.1523/JNEUROSCI.19-14-05875.1999 PMC6783099

[26] Henry GL, Davis FP, Picard S, Eddy SR. Cell type-specific genomics of Drosophila neurons. Nucleic Acids Res. 2012 10;40(19):9691–704. 10.1093/nar/gks671 22855560 PMC3479168

[27] Schilling T, Borst A. Local motion detectors are required for the computation of expansion flow-fields. Biol Open. 2015 7 31;4(9):1105–8. 10.1242/bio.012690 26231626 PMC4582123

[28] Lee T, Luo L. Mosaic analysis with a repressible cell marker for studies of gene function in neuronal morphogenesis. Neuron. 1999 3;22(3):451–61. 10.1016/S0896-6273(00)80701-1 10197526

[29] Diegelmann S, Bate M, Landgraf M. Gateway cloning vectors for the LexA-based binary expression system in Drosophila. Fly (Austin). 2008 Jul-Aug;2(4):236–9. 18776741 10.4161/fly.6817 PMC2913122

[30] Kim D, Pertea G, Trapnell C, Pimentel H, Kelley R, Salzberg SL. TopHat2: accurate alignment of transcriptomes in the presence of insertions, deletions and gene fusions. Genome Biol. 2013 4 25;14(4):R36 10.1186/gb-2013-14-4-r36 23618408 PMC4053844

[31] Liao Y, Smyth GK, Shi W. featureCounts: an efficient general-purpose program for assigning sequence reads to genomic features. Bioinformatics. 2014 4 1;30(7):923–30. 10.1093/bioinformatics/btt656 24227677

[32] Liu WW, Wilson RI. Glutamate is an inhibitory neurotransmitter in the Drosophila olfactory system. Proc Natl Acad Sci U S A. 2013 6 18;110(25):10294–9. 10.1073/pnas.1220560110 23729809 PMC3690841

[33] Mauss AS, Meier M, Serbe E, Borst A. Optogenetic and pharmacologic dissection of feedforward inhibition in Drosophila motion vision. J Neurosci. 2014 2 5;34(6):2254–63. 10.1523/JNEUROSCI.3938-13.2014 24501364 PMC6608528

[34] Mauss AS, Pankova K, Arenz A, Nern A, Rubin GM, Borst A. Neural circuit to integrate opposing motions in the visual field. Cell. 2015 7 16;162(2):351–62. 10.1016/j.cell.2015.06.035 26186189

[35] Srivastava DP, Yu EJ, Kennedy K, Chatwin H, Reale V, Hamon M, et al Rapid, nongenomic responses to ecdysteroids and catecholamines mediated by a novel Drosophila G-protein-coupled receptor. J Neurosci. 2005 6 29;25(26):6145–55. 10.1523/JNEUROSCI.1005-05.2005 15987944 PMC6725065

[36] Buckingham SD, Kidd JF, Law RJ, Franks CJ, Sattelle DB. Structure and function of two-pore-domain K+ channels: contributions from genetic model organisms. Trends Pharmacol Sci. 2005 7;26(7):361–7. 10.1016/j.tips.2005.05.003 15939489

[37] Brand AH, Perrimon N. Targeted gene expression as a means of altering cell fates and generating dominant phenotypes. Development. 1993 6;118(2):401–15. 8223268 10.1242/dev.118.2.401

[38] Lai SL, Lee T. Genetic mosaic with dual binary transcriptional systems in Drosophila. Nat Neurosci. 2006 5;9(5):703–9. 10.1038/nn1681 16582903

[39] Delmas P, Brown DA. Pathways modulating neural KCNQ/M (Kv7) potassium channels. Nat Rev Neurosci. 2005 11;6(11):850–62. 10.1038/nrn1785 16261179

[40] Wei A, Covarrubias M, Butler A, Baker K, Pak M, Salkoff L. K+ current diversity is produced by an extended gene family conserved in Drosophila and mouse. Science. 1990 5 4;248(4955):599–603. 10.1126/science.2333511 2333511

[41] Srinivasan S, Lance K, Levine RB. Contribution of EAG to excitability and potassium currents in Drosophila larval mo toneurons. J Neurophysiol. 2012 5;107(10):2660–71. 10.1152/jn.00201.2011 22323637 PMC3362287

[42] Martinson AS, van Rossum DB, Diatta FH, Layden MJ, Rhodes SA, Martindale MQ, et al Functional evolution of Erg potassium channel gating reveals an ancient origin for IKr. Proc Natl Acad Sci U S A. 2014 4 15;111(15):5712–7. 10.1073/pnas.1321716111 24706772 PMC3992637

[43] Enell L, Hamasaka Y, Kolodziejczyk A, Nässel DR. gamma-Aminobutyric acid (GABA) signaling components in Drosophila: immunocytochemical localization of GABA(B) receptors in relation to the GABA(A) receptor subunit RDL and a vesicular GABA transporter. J Comp Neurol. 2007 11 1;505(1):18–31. 10.1002/cne.21472 17729251

[44] Tritsch NX, Oh WJ, Gu C, Sabatini BL. Midbrain dopamine neurons sustain inhibitory transmission using plasma membrane uptake of GABA, not synthesis. Elife. 2014 4 24;3:e01936 10.7554/eLife.01936 24843012 PMC4001323

[45] Yoon BE, Woo J, Chun YE, Chun H, Jo S, Bae JY, et al Glial GABA, synthesized by monoamine oxidase B, mediates tonic inhibition. J Physiol. 2014 11 15;592(22):4951–68. 10.1113/jphysiol.2014.278754 25239459 PMC4259537

[46] Kim JI, Ganesan S, Luo SX, Wu YW, Park E, Huang EJ, et al Aldehyde dehydrogenase 1a1 mediates a GABA synthesis pathway in midbrain dopaminergic neurons. Science. 2015 10 2;350(6256):102–6. 10.1126/science.aac4690 26430123 PMC4725325

[47] Bessman SP, Rossen J, Layne EC. Gamma-Aminobutyric acid-glutamic acid transamination in brain. J Biol Chem. 1953 3;201(1):385–91. 13044808

[48] Balazs R, Machiyama Y, Hammond BJ, Julian T, Richter D. The operation of the gamma-aminobutyrate bypath of the tricarboxylic acid cycle in brain tissue in vitro. Biochem J. 1970 2;116(3):445–61. 5435689 10.1042/bj1160445 PMC1185383

[49] O'Malley DM, Sandell JH, Masland RH. Co-release of acetylcholine and GABA by the starburst amacrine cells. J Neurosci. 1992 4;12(4):1394–408. 1556600 10.1523/JNEUROSCI.12-04-01394.1992 PMC6575809

[50] Ding H, Smith RG, Poleg-Polsky A, Diamond JS, Briggman KL. Species-specific wiring for direction selectivity in the mammalian retina. Nature. 2016 7 7;535(7610):105–10. 10.1038/nature18609 27350241 PMC4959608

